# Dietary Supplementation with Compound Probiotics and Berberine Alters Piglet Production Performance and Fecal Microbiota

**DOI:** 10.3390/ani10030511

**Published:** 2020-03-19

**Authors:** Xiaoxiang Xu, Canyu Yang, Juan Chang, Ping Wang, Qingqiang Yin, Chaoqi Liu, Tianzeng Gao, Xiaowei Dang, Fushan Lu

**Affiliations:** 1 College of Animal Science and Veterinary Medicine, Henan Agricultural University, Zhengzhou 450002, China; Xiaoxiangxu@stu.henau.edu.cn (X.X.); 18838918005@163.com (C.Y.); changjuan2000@henau.edu.cn (J.C.); wangping@henau.edu.cn (P.W.); liuchaoqi2018@henau.edu.cn (C.L.); 2 Henan Guangan Biotechnology Co. Ltd., Zhengzhou 450001, China; zheng196604@126.com; 3 Henan Delin Biological Product Co., Ltd., Xinxiang 453000, China; dang355@163.com; 4 Henan Puai Feed Co., Ltd., Zhoukou 466000, China; lufushan@puaifeed.com

**Keywords:** piglets, compound probiotics, berberine, fecal microbiota, production performance

## Abstract

**Simple Summary:**

In order to find antibiotic substitutes for weaned piglet health and growth, compound probiotics and berberine (CPB) were selected in this study. The results indicated that CPB could replace antibiotics to improve piglet health and decrease mortality, diarrhea and rejection rates. CPB was also able to regulate fecal microbiota as well as improve protein digestibility and serum biochemical parameters. Therefore, CPB might be a good antibiotic alternative in piglet production performance.

**Abstract:**

This study was conducted to investigate the effects of dietary supplementation with compound probiotics and berberine (CPB) on growth performance, nutrient digestibility and fecal microflora in weaned piglets. A total of 200 piglets 35 days old were randomly allocated to 5 groups, 4 replications in each group, and 10 piglets in each replication. Group A was the basal diet; group B was supplemented with antibiotics and zinc oxide; groups C, D and E were supplemented with 0.06%, 0.12% and 0.18% CPB, respectively. The experimental period was 42 d. The results indicated that there were no significant differences in average daily feed intake (ADFI), average daily gain (ADG) and feed conversion rate (FCR) among five groups (*p* > 0.05). However, mortality, diarrhea and rejection rates in the control group were higher than that in other groups. CPB could increase protein digestibility and serum IgG content (*p* < 0.05), while it could decrease serum urea nitrogen content and alkaline phosphatase activity (*p* < 0.05). Analysis of fecal microbiota showed that the relative abundances of *Bacteroides* and *Firmicutes* were increased, while the relative abundances of opportunistic pathogens such as *Spirochaetae* and *Protebactreria* were dramatically decreased in piglets fed with CPB or antibiotics, compared with the control group. Furthermore, CPB intervention increased the relative abundances of *Prevotella_9, Megasphaera* and *Prevotella_2*, while decreased the relative abundance of *Prevotellaceae_NK3B31_group*. Correlation analysis revealed that there was good correlation between serum indexes and fecal microbiota. It was suggested that CPB might be a promising antibiotic alternative for improving piglet health and immunity, decreasing mortality by positively altering gut microbiota.

## 1. Introduction

Growth-promoting antibiotics have widely been used to improve animal growth and prevent post-weaning diarrhea for weaned pigs. However, routine use of antibiotics is debatable in current society because of the horizontal transfer of antibiotic-resistance genes from bacteria in farm animals to human food [1] as well as the growing number of antibiotic-resistant bacteria. In spite of the fact that antibiotics have demonstrated an improvement in growth performance, their long-term use may enrich the population of potential pathogenic bacteria to cause negative effects on the host [2]. However, reducing antibiotic use will increase post-weaning diarrhea, mortality and feeding costs in swine production. Therefore, natural and safe alternatives with similar activities are in urgent need of development, which will improve animal health, maintain bio-safety and have low residue. Since the use of in-feed antibiotic growth promoters (AGPs) has been banned in European and other countries, probiotics and Chinese herbal medicine extracts have been considered as the good alternatives to improve animal health and protect against infectious challenges [3].

Probiotics are proposed as alternatives to sub-therapeutic antibiotics in livestock industry, which play very important roles in promoting digestion, improving intestinal immune status and maintaining microbial balance in piglets. Previous studies have showed that probiotics can improve growth performance and prevent disease by boosting immune function and stimulating the host’s mucosal immune system [4,5]. *Lactobacillus frumenti*, as a predominant bacterium in intestine of weaned piglets, can improve intestinal epithelial barrier functions in early weaned piglets [6]. Dietary supplementation of yeast cultures and yeast products can improve immune function and intestinal development in weaned piglets [7]. Additionally, compound probiotics have been suggested to have greater efficacy for host than the single-strain types [8]. Many reports have demonstrated that compound probiotics have significant functions for resisting hypertension, hyperglycemia, cancer, oxidation, inflammatory and hypolipidemia, as well as promoting the proliferation of beneficial bacteria and inhibiting the reproduction of harmful bacteria as a feed additive. Therefore, the compound probiotics including *Lactobacillus casei* (*L. casei*), *Enterococcus faecalis* (*E. faecalis*), *Bacillus subtilis* (*B. subtilis*) and *Saccharomyces cerevisiae boulardii* (*S. cerevisiae boulardii*) were used to improve weaned piglet health and growth in the current study.

Berberine is one kind of quaternary benzylisoquinoline plant alkaloid with a proven medicinal history in Ayurvedic and Chinese medicinal systems [9]. Berberine as an active constituent in the root, rhizome and stem bark of many medicinally important plants has demonstrated a wide spectrum of pharmacological effects [10,11]. Another study indicated that dietary supplementation with probiotics-fermented *Massa Medicata Fermentata* could enhance host intestinal homeostasis by modulating the composition of gut microbiota to ameliorate the weaning stress in piglets [12]. Therefore, it is supposed that dietary supplementation with compound probiotics and berberine can regulate gut microbiota as well as improve growth performance and intestinal immunity, which may have a great potential value for its development and application in animal production.

## 2. Materials and Methods

### 2.1. Compound Probiotics and Berberine Preparation

Based on the previous research in our laboratory, the compound probiotics used in this study were composed of *L. casei*, *E. faecalis*, *B. subtilis* and *S. cerevisiae boulardii* at a ratio of 1:1:1:1, which contained the viable counts of 1 × 10^9^, 1 × 10^10^, 1 × 10^9^, 1 × 10^10^ CFU/g, respectively. Berberine with the purity of 98% was kindly provided by Henan Delin Biological Products Co. Ltd. (Xinxiang, China). The compositions of compound probiotics and berberine (CPB) were 0.29% *L. casei*, 33.69% *E. faecalis*, 9.43% *B. subtilis*, 47.16% *S. cerevisiae boulardii* and 9.43% berberine based on the inhibiting-bacterium result obtained in our laboratory.

### 2.2. Animals Diets and Managements

A total of 200 weaned piglets 35 days old (Duroc × Landrace × Large White) with an initial body weight of 9.58 ± 0.79 kg were randomly allocated to 5 groups, 4 replications in each group, and 10 piglets (half castrated male and half female) in each replication. Group A was the basal diet; group B was supplemented with antibiotics (quetiapine ketene, calcium terramycin and methylene salicylic acid) and zinc oxide (1600 mg/kg); and groups C, D and E were supplemented with 0.06%, 0.12% and 0.18% CPB, respectively. All piglets were housed in slatted floor indoor pens under standard conditions. The experimental period was 42 d. Feed and water were supplied ad libitum. The shed temperature was 23 ± 2 °C, and the relative humidity was maintained at 55%–60%. The feeding management and immune procedure were conducted according to the standard requirements in the pig farm. All the animals were managed according to the guidelines for the care and use of experimental animals approved by The Ethics Committee of Henan Agricultural University (SKLAB-B-2010-003-01). The diet compositions and nutrient levels are listed in Table 1.

### 2.3. Sample Measurements

Body weights of each piglet were measured on the first and last day of the experiment. The feed intake was measured daily. The average daily gain (ADG), average daily feed intake (ADFI), feed conversion rate (FCR), diarrhea date, mortality and rejection rates were calculated during the experiment.

Fecal samples were respectively taken without contamination from each of 5 castrated piglets in each group for 3 d at the end of experiment. The individual 3-day fecal sample of each castrated piglet was mixed, selected and stored at −20 °C for further analysis. Some of the fecal samples were dried at 65 °C and mashed to determine nutrient digestibility. Crude protein (CP), ether extract (EE), calcium (Ca) and phosphorus (P) in diets and feces were measured with Kjeldahl, ether extract, potassium permanganate (KMnO4) and ammonium molybdate ((NH_4_)_6_Mo_7_O_24_) protocols, respectively. The 4 N hydrochloric acid insoluble ashes in diets and feces were used as an indicator to calculate the nutrient digestibility with the following formula: Nutrient digestibility = 100 − (indicator content in diet / nutrient content in diet × nutrient content in feces / indicator content in feces) × 100.

About 5 mL blood samples were collected from the precaval veins of 3 castrated piglets in each group. After the blood was tilted at room temperature for 3 h, the serum was collected by transferpettor and stored in a centrifuge tube at −20 °C for further analysis. The serum biochemical parameters such as urea nitrogen (UN), glucose (GLU), total protein (TP), alanine aminotransferase (ALT), aspartate aminotransferase (AST), alkaline phosphatase (ALP), immunoglobulin G (IgG) and immunoglobulin M (IgM) were measured with a 7600-020 Automatic Analyzer (Hitachi Ltd., Tokyo, Japan) in the Biochemical Laboratory of Zhengzhou University, Zhengzhou, China.

### 2.4. DNA Extraction and 16S rRNA Sequencing for Fecal Microbial Community Ananlysis

To investigate gut microbiota in the feces of weaned piglets, fecal samples were collected from 3 piglets in Groups A, B, and C, respectively. The total genomic DNA was extracted from fecal sample using Soil DNA Kit (Omega Biotek, Norcross, GA, USA) according to the manufacturer’s instructions. The final DNA concentration and purity were investigated by NanoDrop 2000 UV–vis spectrophotometer (Thermo Scientific, Wilmington, NC, USA), and DNA quality was checked by 1% agarose gel electrophoresis. The V3-V4 region of the 16S rRNA gene was amplified with 338F up-stream primer (5′-ACTCCTACGGGAGGCAGCAG-3′) and 806R down-stream primer (5′-GGACTACHVGGGTWTCTAAT-3′) by PCR (GeneAmp 9700, ABI, USA). The PCR amplification program was set as follows: an initial denaturation at 95 °C for 3 min; 28 cycles of 95 °C for 30 s, annealing at 55 °C for 30 s, elongation at 72 °C for 45 s; and a final extension at 72 °C for 10 min. PCR reactions were carried out in 20 µL reaction mixture containing 10 ng template DNA, 4 µL 5× FastPfu buffer, 2 µL 2.5 mM dNTPs, 0.8 µL each primer (5 µM), and 0.4 µL FastPfu polymerase. The amplified PCR products were extracted by 2% agarose gel, purified by the AxyPrep DNA Gel Extraction Kit (Axygen Biosciences, Union City, CA, USA), and further quantified using QuantiFluorTM-ST (Promega (Beijing) Biotech Co., Ltd. Beijing, China) according to the manufacturer’s instructions. The purified amplification products were pooled in equimolar amounts and sequenced on an Illumina MiSeq platform (Illumina, San Diego, CA, USA).

### 2.5. Bioinformatics Analysis of Sequencing Data

Raw sequencing reads were demultiplexed and quality-filtered by Trimmomatic with the following criteria: (1) the 300-bp reads were truncated at any site receiving an average quality score < 20 over a 50-bp sliding window; (2) primers matching exactly allowed two-nucleotide primer mismatches, and reads containing ambiguous bases were removed; (3) sequences whose overlap was longer than 10 bp were assembled, and the reads that could not be assembled were discarded. Operational taxonomic units (OTUs) were clustered using UPARSE (version 7.11, http://drive5.com/uparse/) with a cutoff of 97% similarity, and the chimeric sequences were identified and removed using UCHIME. Taxonomic classification of phylotypes was analyzed by RDP v2.2 Classifier program against the Silva (SSU123) 16S rRNA database with a 70% confidence threshold. Biodiversity of the samples was calculated with Species Richness Estimator (ACE and Chao), diversity indices (Shannon and Simpson), Shannon evenness (a Shannon index-based evenness), and Good’s coverage. The analysis of molecular variance (AMOVA) was performed by comparing the differences among three groups. The results of 16S rRNA gene sequencing were applied by I-sanger platform (http://www.i-sanger.com) in order to predict species annotation, composition, differences, and functional prediction of the bacterial community in the fecal samples of piglets. Venn diagrams were used to evaluate the distribution of OTUs among the different groups. Un-weighted UniFrac distances were measured to determine the difference in microbial communities.

### 2.6. Statistical Analysis

Statistical analyses were performed using SPSS Statistics Software (version 18.0, IBM, New York, NY, United States). Data were evaluated by one-way ANOVA, and the comparative analysis was conducted by using the method of Duncan’s test. Statistical results were shown in mean ± SEM, and *P* < 0.05 was considered as statistical significance.

## 3. Results

### 3.1. Growth Performance, Nutrient Digestibility and Serum Parameters

Table 2 indicated that CPB had no significant effects on ADG, ADFI and FCR, compared with the control and antibiotic groups (*p* > 0.05). However, the mortality, diarrhea and rejection rates of piglets in the control group were higher than that in other groups, indicating that CPB was probably a good alternative to antibiotics. Table 3 showed that CPB could significantly increase protein digestibility (*p* < 0.05), compared with the control and antibiotic groups. Table 4 showed that serum IgG concentration in group D was higher than that in group A (*p* < 0.05), serum UN concentration in group C and D were lower than that in group B (*p* < 0.05). Compared with group B, serum ALT and ALP concentrations in group A and AST in group C were significantly increased (*p* < 0.05). There were no significant differences for serum GLU, TP and IgM contents among the five groups (*p* > 0.05).

### 3.2. Sequencing Data

**Figure 1:**
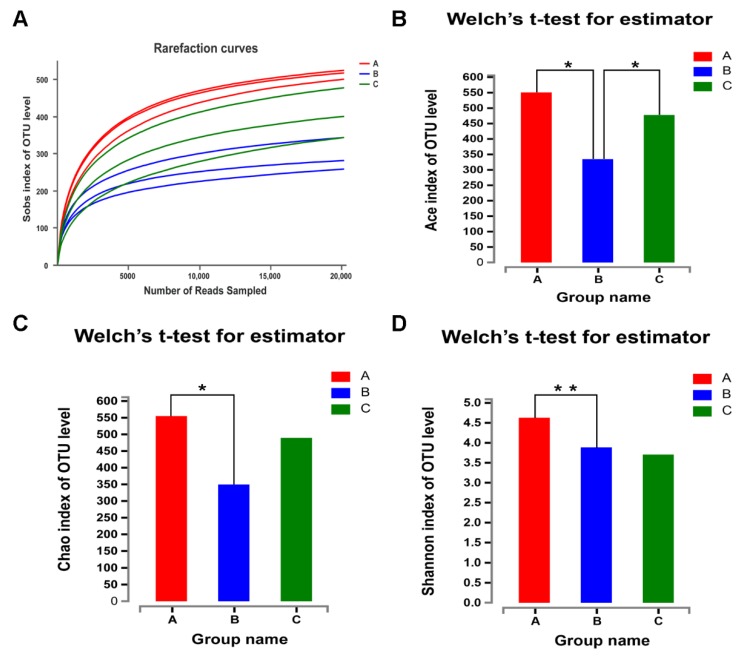
Rarefaction curves and different microbial diversity indices in groups A, B and C. (**A**) Rarefaction curves of the operational taxonomic units (OTUs) number at 97% similarity box plot for every sample. (**B**–**D**) Different microbial diversity indexes such as Ace, Chao and Shannon among groups A, B and C. The ACE, Chao1 and Shannon indexes are presented for a similarity of 97% between reads. * *p* ≤ 0.05, ** *p* ≤ 0.01. Group A: basal diet; group B: basal diet supplemented with antibiotics and zinc oxide; group C: basal diet supplemented with 0.06% CPB.

The V3-V4 region of 16S rRNA gene was respectively sequenced from nine fecal samples (three samples for groups A, B and C) by the Illumina Miseq high-throughput sequencing platform. After removing incorrect and chimeric sequences, a total of 424,914 high-quality reads were generated. An average of 47,212 sequences per sample were obtained with an average length of 441 bp. A total of 1540 OTUs were identified using the criterion of 97% sequence similarity at the species level, all of them belonged to the bacterial domain according to the Greengenes classification. Finally, 513 OTUs (Good’s coverage) per sample were identified. Figure 1A showed that this sequencing range was sufficient to cover the microbial diversity of each sample. Sequence information and calculated microbial diversity indices of the fecal samples were presented in Figure 1B–D. The ACE indexes in group A and group C were higher than that in group B (*p* < 0.05). The Chao1 and Shannon indexes in group A were higher than that in group B (*p* < 0.05), indicating that antibiotics decreased fecal microbial diversity and richness.

### 3.3. Diversity and Compositions of Fecal Microbiota

**Figure 2:**
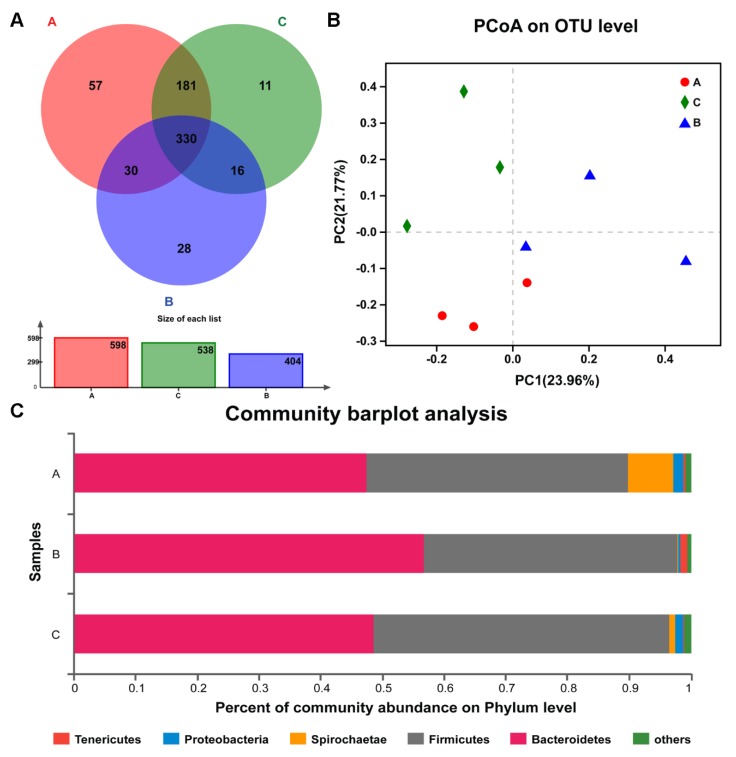
Venn and Principal Coordinates Analysis (PCoA) analyses for bacterial composition in piglet feces. (**A**) Venn diagrams for bacterial OTUs in groups A, B and C. (**B**) Principal coordinate analysis based on unweighted UniFrac metrics in groups A, B and C. (**C**) Fecal bacterial composition calculated at the phylum level. The different colors represent the relative abundance of bacteria in three groups. Group A: basal diet; group B: basal diet supplemented with antibiotics and zinc oxide; group C: basal diet supplemented with 0.06% CPB.

Figure 2A indicated the total number of OTUs in group A was higher than other groups. In addition, a total of 330 OTUs were shared by the three groups, which mostly belonged to *Bacteroidetes*, *Firmicutes*, and *Proteobacteria* phyla. Furthermore, the CPB group had the lowest number of unique OTUs among the three groups. Principal Coordinates Analysis (PCoA) analysis of bacterial OTUs based on the un-weighted UniFrac distance metrics revealed that the compositions of microbial communities had significant differences among three groups (Figure 2B). The samples in group A were clustered closely, indicating that their bacterial community structures were highly similar and stable, while the samples in groups B and C were clustered loosely. A total of 17 phyla, 27 classes, 67 families and 187 genera were identified from 9 fecal samples of piglets. At the phylum level (Figure 2C), *Bacteroides* and *Firmicutes* were the dominant bacteria with high relative abundances accounting for 47.39%–56.7% and 41.23%–48.09% respectively, which was higher in group B and group C than that in group A. However, the relative abundance of *Spirochaetae* was lower in group B and C than that in group A.

**Figure 3:**
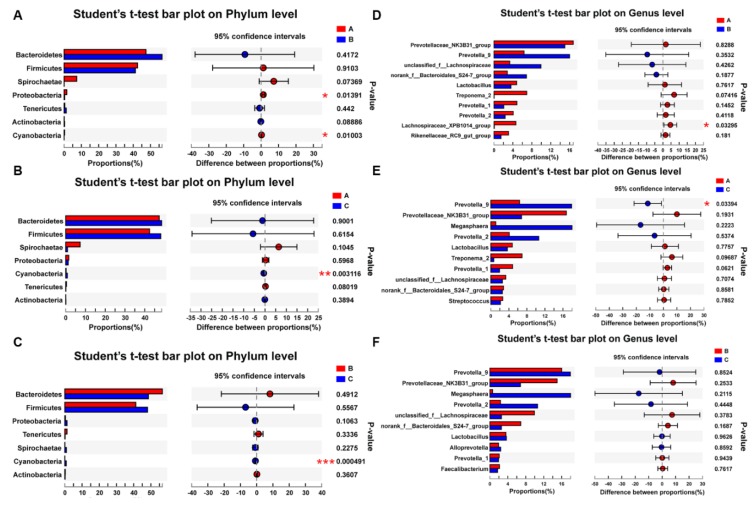
The different analyses of bacterial community at the phylum and genera levels. (**A**–**C**) The difference in bacterial community on the phylum level by Student’s t-test between groups A and B, between groups A and C, and between groups B and C, respectively. (**D**–**F**) The difference in bacterial community on the genera level by Student’s t-test between groups A and B, between groups A and C, and between groups B and C, respectively. * *p* ≤ 0.05, ** *p* ≤ 0.01, *** *p* ≤ 0.001. Group A: basal diet; group B: basal diet supplemented with antibiotics and zinc oxide; group C: basal diet supplemented with 0.06% CPB.

**Figure 4:**
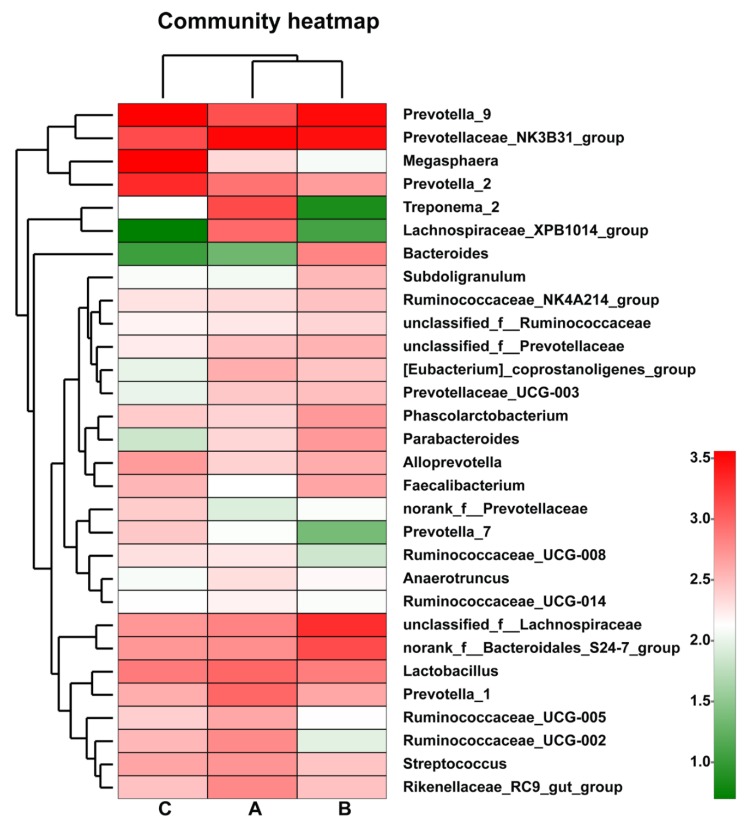
Microbial community heatmap of top 30 bacteria on the genera level. The different colors represent the relative abundance of bacteria in groups A, B and C. The red color means higher relative abundance, whereas the green color means lower relative abundance. Group A: basal diet; group B: basal diet supplemented with antibiotics and zinc oxide; group C: basal diet supplemented with 0.06% CPB.

Figure 3A–C showed that the relative abundance of *Cyanobacteria* in groups B and C was increased significantly, compared with group A (*p* < 0.05). Furthermore, the relative abundances of *Spirochaetae*, *Proteobacteria*, *Tenericutes* and other bacteria were lower among the three groups. The abundance of *Lachnospiraceae_XPB1014_group* was decreased significantly in the antibiotic group (*p* < 0.05), compared with the control group (Figure 3D–F). The abundances of top 30 bacterial communities in feces at the genus level were presented in the hierarchically clustered heatmap (Figure 4). *Prevotella* was the dominant bacteria in all the groups, and its dominant position was not changed by feeding with CPB or antibiotics. The relative abundances of *Prevotella_9*, *Megasphaera* and *Prevotella_2* in the CPB group were increased, compared with the control group (*p* < 0.05); however, the relative abundance of *Prevotellaceae_NK3B31_group* in the CPB group was lower than that in the control and antibiotics groups.

### 3.4. Differences in Bacterial Communities

**Figure 5:**
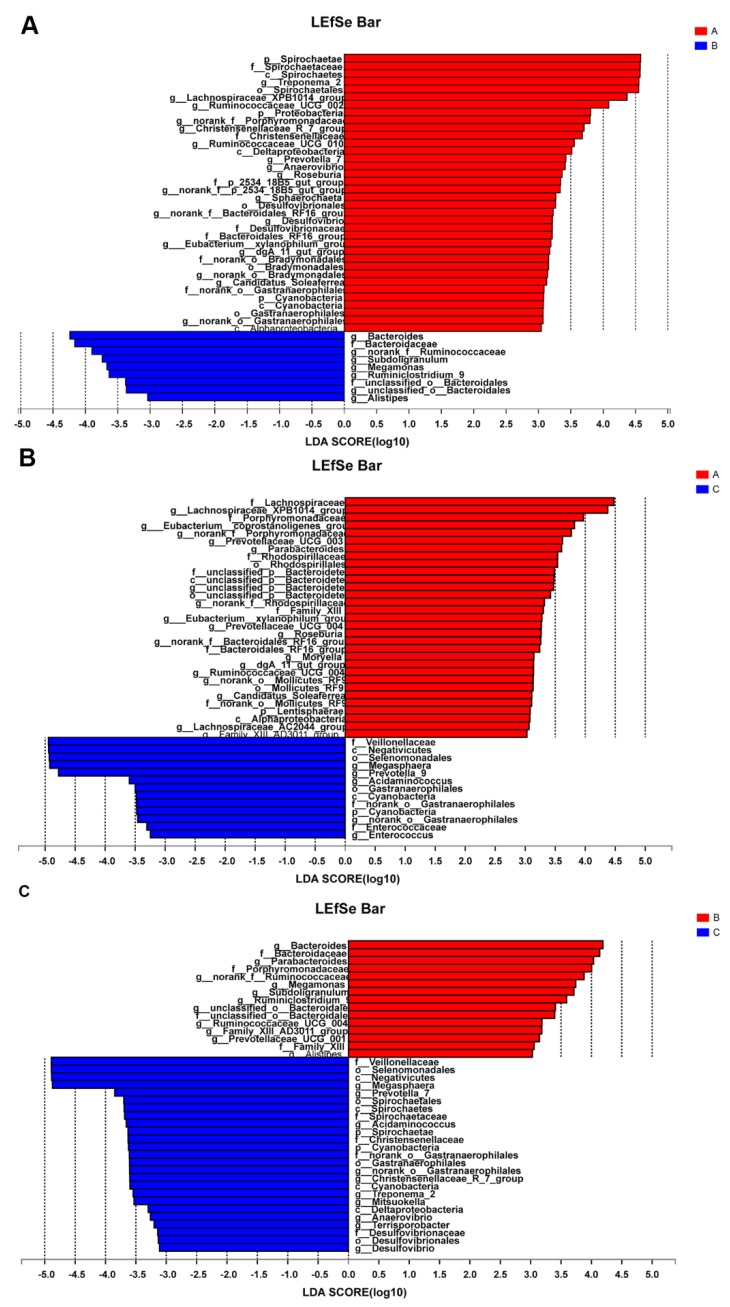
Linear Discriminant Analysis (LDA) on the genus level. Bacterial taxa at the genus level significantly identified by linear discriminant analysis coupled with effect size (LEfSe) using the default parameters between groups A and B (**A**), between groups A and C (**B**), and between groups B and C (**C**), respectively. Group A: basal diet; group B: basal diet supplemented with antibiotics and zinc oxide; group C: basal diet supplemented with 0.06% CPB.

To identify the significant abundant bacterial taxa in response to the different treatments, the OTUs of each group were compared, and LEfSe with a three-threshold value of Linear Discriminant Analysis (LDA) at the genus level was performed to detect the biomarkers. The results showed that there were 36 taxa in group A and 9 taxa in group B (Figure 5A), 31 taxa in group A and 13 taxa in group C (Figure 5B), and 15 taxa in group B and 25 taxa in group C (Figure 5C), suggesting that the gut microbiota may be altered by feeding with CPB or antibiotics in piglets.

### 3.5. Association and Model Predictive Analysis

**Figure 6:**
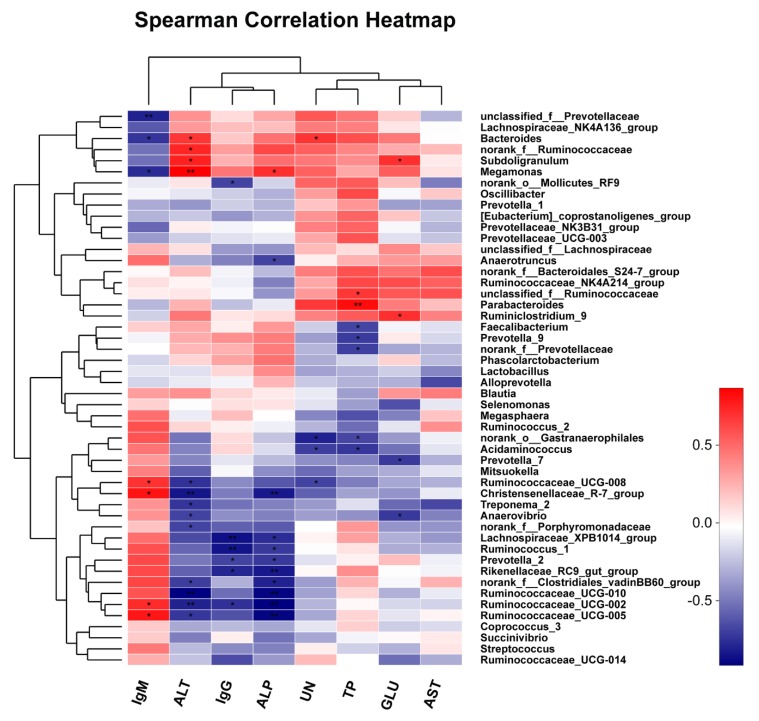
Heatmap of the correlation analysis between the top 50 bacterial genera and the environmental factors. UN: urea nitrogen, GLU: glucose, TP: total protein, IgM: immunoglobin M, IgG: immunoglobin G, ALT: alanine aminotransferase, AST: aspartate aminotransferase, ALP: alkaline phosphatase. * *p* ≤ 0.05, ** *p* ≤ 0.01. Group A: basal diet; group B: basal diet supplemented with antibiotics and zinc oxide; group C: basal diet supplemented with 0.06% CPB.

Correlation analysis was conducted between the serum indices and the top 50 bacterial genera, which was directly reflected through a heatmap (Figure 6). The threshold |R| > 0.4 was considered as being correlated. The results indicated that *Ruminococcaceae_UCG-008*, *Christensenellacease_R-7_group*, *Ruminococcaceae_UCG-002* and *Ruminococcaceae_UCG-005* were positively correlated with IgM, while *Bacteroides*, *Megamonas* and *unclassified_f_Prevotellaceae* were negatively correlated with IgM. *Bacteroides*, *Megamonas*, *Subdoligranulum* and *norank_f_Ruminococcaceae* were positively correlated with ALT, while *Ruminococcaceae_UCG-010*, *Treponema_2*, *Christensenellaceae_R-7_group*, *Ruminococcaceae_UCG-002*, *Anaerovibrio*, *Ruminococcaceae_UCG-008*, *Ruminococcaceae_UCG-005*, *norank_f_Porphyromonadaceae*, *norank_f_Clostridiales_vadinBB60_group* and *Lachnospiraceae_XPB1014_group* were negatively correlated with ALT. *Lachnospiraceae_XPB1014_group*, *Rikenellaceae_RC9_gut_group*, *Ruminococcus_1*, *Ruminococcaceae_UCG-002*, *norank_o_Mollicutes_RF9* and *Prevotella_2* were negatively correlated with IgG. *Bacteroides* was positively correlated with UN, while *Acidaminococcus*, *norank_o_Gastranaerophilales* and *Ruminococcaceae_UCG-008* were negatively correlated with UN. *Megamonas* was positively correlated with ALP, while *Ruminococcaceae_UCG-002*, *Ruminococcaceae_UCG-010*, *Ruminococcus_1*, *Ruminococcaceae_UCG-005*, *Christensenellaceae_R-7_group*, *Anaerotruncus*, *Rikenellaceae_RC9_gut_group*, *norank_f_Clostridiales_vadinBB60_group*, *Lachnospiraceae_XPB1014_group* and *Prevotella_2* were negatively correlated with ALP. *Parabacteroides* and *unclassified_f_Ruminococcaceae* were positively correlated with TP, while *norank_o_Gastranaerophilales*, *Faecalibacterium*, *norank_f_Prevotellaceae*, *Prevotella_9* and *Acidaminococcus* were negatively correlated with TP. *Ruminiclostridium_9* and *Subdoligranulum* were positively correlated with GLU, while *Anaerovibrio* and *Prevotella_7* were negatively correlated with GLU. However, there was no correlation between microbiota and AST.

### 3.6. Predicted Functional Profiles of Microbial Communities Using PICRUSt

**Figure 7:**
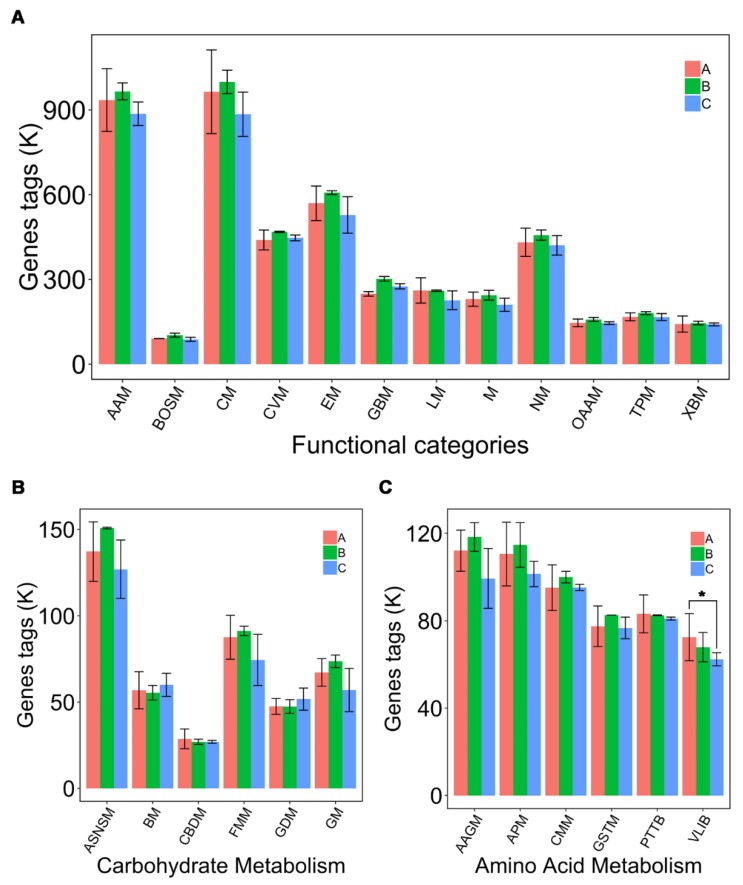
Prediction for Kyoto Encyclopedia of Genes and Genomes (KEGG) pathway of bacterial communities using PICRUSt program. (**A**) Prediction for metabolism of bacterial communities. AAM, amino acid metabolism; BOSM, biosynthesis of other secondary metabolites; CM, carbohydrate metabolism; CVM, metabolism of cofactors and vitamins; EM, energy metabolism; GBM, glycan biosynthesis and metabolism; LM, lipid metabolism; M, metabolism; NM, nucleotide metabolism; OAAM, metabolism of other amino acids; TPM, metabolism of terpenoids and polyketides; XBM, xenobiotics biodegradation. (**B**) Prediction for carbohydrate and amino acid metabolisms of bacterial communities. ASNSM, amino sugar and nucleotide sugar metabolism; BM, butanoate metabolism; CBDM, C5-Branched dibasic acid metabolism; FMM, fructose and mannose metabolism; GDM, glyoxylate and dicarboxylate metabolism; GM, galactose metabolism. (**C**) Prediction for amino acid metabolism of bacterial communities. AAGM, alanine, aspartate and glutamate metabolism; APM, arginine and proline metabolism; CMM, cysteine and methionine metabolism; GSTM, glycine, serine and threonine metabolism; PTTB, phenylalanine, tyrosine and tryptophan biosynthesis; VLIB, valine, leucine and isoleucine biosynthesis. * *p* ≤ 0.05. Group A: basal diet; group B: basal diet supplemented with antibiotics and zinc oxide; group C: basal diet supplemented with 0.06% CPB.

To predict the potential functional profiles of gut bacteria in nutrient metabolism for piglets fed with CPB or antibiotics, Kyoto Encyclopedia of Genes and Genomes (KEGG) pathways were analyzed by PICRUSt program. Biological pathways were organized in six functional categories including metabolism, cellular processes, environmental information processing, genetic information processing, human diseases and organism systems to compare the functional enrichment in each group (Table 5). Carbohydrate and amino acid metabolisms were the two main metabolic pathways in the metabolic functional category (Figure 7A). A total of 252 KEGG pathways were predicted in three groups. Six pathways related to carbohydrate metabolism (Figure 7B) and six pathways related to amino acid metabolism (Figure 7C) were the focus, which included the metabolisms of amino sugar, nucleotide sugar, butanoate, C5-branched dibasic acid, fructose, mannose, galactose, glyoxylate, dicarboxylate, valine, glycine, serine, threonine, alanine, aspartate, glutamate, arginine, proline, cysteine, methionine, phenylalanine, leucine, isoleucine, tyrosine and tryptophan biosyntheses. The number of genes related to valine, leucine and isoleucine biosynthesis pathway in the CPB group were significantly decreased compared with the control group (*p* < 0.05).

## 4. Discussion

A large number of microorganisms inhabit in the intestines of animals. The host provides sufficient nutrients and a stable space environment for the intestinal microbiota, and the microorganisms participate in nutrient digestion and absorption as well as regulate the physiological functions for the host. After weaning, the piglets will be threatened by the challenges from diets, environmental stress and harmful bacteria, which contributes to dysfunctions of intestinal and immune system, disrupts gut microbial ecosystem, weakens nutrient digestion and adsorption, inhibits growth performance and health of piglets. Currently, antibiotics play important roles in preventing early-weaning stress-induced intestinal epithelial barrier function damage and post-weaning diarrhea in piglets [13]. However, antibiotics will be banned around the world due to their side-effects. Therefore, the study on alternatives to antibiotics becomes more and more important. Numerous studies have demonstrated that probiotics can promote the proliferation of beneficial bacteria in animal intestines for improving animal health [6,14]. In addition, berberine has abundant pharmacological functions [9]. Therefore, the optimal mixtures of probiotics and berberine may be a good alternative to antibiotics. This result indicated that dietary supplementation with antibiotics or CPB had no significant effect on piglet growth even though protein digestibility was improved by CPB addition, in agreement with the previous report in which dietary supplementation with *Clostridium butyricum* for 28 d could improve feed efficiency but without significant effect on ADG and ADFI for piglets [15]; however, mortality, diarrhea and rejection rates were decreased by antibiotics or CPB addition. It seems to be that CPB is better than antibiotics for reducing mortality, indicating that CPB should be a good alternative to probiotics. The reason may be from the improvement of intestinal environment and the promotion of beneficial microorganism proliferations.

The serum biochemical indexes can provide evidence about the health and metabolism for animals. AST, ALT and ALP exist inside cells, which will be released into the serum when the cells are damaged. It was reported that the probiotics have the capacity to modulate animal immune system by enhancing the systematic antibody response to soluble antigens in serum [16]. In this study, the lower serum activities of ALT, ALP and AST in the probiotic group indicated that antibiotics had a certain protective effect on cell function for piglets. The high IgG concentration in CPB group may be from gut lymphocytes stimulated by probiotics in agreement with the previous reports [17,18], which can improve animal immunity and anti-infection ability. The lower serum UN content in groups C and D indicated that protein metabolism was improved by CPB, corresponding with high protein digestibility caused by CPB addition.

In recent years, with the development of sequencing technology, the Illumin Miseq sequencing platform has been used to successfully detect the diversity of piglet intestinal microbiota with more than 454 pyrosequencings and high coverage [19]. The high ACE index indicated that CPB treatment could increase the richness of gut microbiota, indicating that CPB is better than antibiotics for improving gut microbial community. A good gut microbiota is able to benefit the host by regulating physiological process and mucosal immunity such as suppressing proliferation of enteric pathogens [20], strengthening integrity of intestinal barrier on epithelial cells [21,22] and producing antimicrobial peptides in mucus layer [23]. In this study, *Bacteroides* and *Firmicutes* were the two dominant phyla, and *Prevotella* was the dominant bacterial species at the genus level, corresponding with the previous studies in which the most abundant phyla detected in the fecal samples of weaned piglets were *Firmicutes* and *Bacteroides* [24,25,26,27]. This research showed that *Firmicutes* abundance was increased in the CPB group but decreased in the antibiotic group. It was shown that *Firmicutes* was the most abundant phylum in pre-weaning pigs, and this shifted gradually to *Bacteroidetes* after weaning [26], indicating that the dominant phylum will be changed by age, diet, antibiotics, probiotics, environment, and so on. At the genus level, the relative abundances of *Prevotella_9*, *Megasphaera* and *Prevotella_2* were higher, and *Prevotellaceae_NK3B31_group* abundance was lower in the CPB group than that in the control group; however, the relative abundances of *Treponema_2* and *Lachnospiraceae_XPB1014_group* were lower in the CPB and antibiotics groups than that in the control group. The previous report indicated that *Prevotella* became one of the most abundant genera in pig gut after weaning [28], in agreement with this result made by CPB addition. This study suggests that administration of CPB can increase the diversity and abundance of beneficial bacteria and speed up the development and maturation of gut microbial community. In addition, through using PICRUSt program to predict functional profiles of microbial communities, the number of gene tags involved in valine, leucine and isoleucine biosynthesis pathways in the CPB group were significantly decreased compared with control group, indicating that administration of CPB may be involved in amino acid metabolism by altering gut microbiota in piglets.

The composition of the fecal microbiota in weaned pigs is likely to be shaped by environmental factors such as antibiotic use, stress, pen location, nutrient supplements or seasonal effects [29]. In this study, the composition of microbiota at the phylum and genus levels showed multiple variety in three groups. Correlation analysis between the top 50 microbial genera and serum indices revealed that the relative abundances of *Ruminococcaceae_UCG-008*, *Christensenellacease_R-7_group*, *Ruminococcaceae_UCG-002* and *Ruminococcaceae_UCG-005* were positively correlated with IgM and negatively correlated with ALT, ALP, UN in serum; the higher abundances of these genera caused CPB addition and indicated that CPB was better than antibiotics for increasing immunity, protein metabolism and antioxidative ability. However, the relative abundances of *Bacteroides* and *Megamonas* were positively correlated with the ALT, ALP, UN, and negatively correlated with IgM. The high *Bacteroides* abundance by antibiotics addition inferred that antibiotics influenced piglet health. The correlation analysis showed that the relative abundances of *Lachnospiraceae_XPB1014_group*, *Rikenellaceae_RC9_gut_group*, *Ruminococcaceae_UCG-002* and *Prevotella_2* were negatively correlated with serum IgG content; the lower abundances of these genera triggered by CPB addition indicated that CPB was able to increase host immunity, corresponding with the high serum IgG concentration caused by CPB administration. The previous report showed that probiotics could increase host serum IgG levels [30,31], in agreement with this study. This study demonstrated that oral administration of CPB enhanced host intestinal homeostasis by modulating the composition of gut microbiota for improving piglet production performance.

## 5. Conclusions

This study showed that dietary supplementation with CPB instead of antibiotics could improve piglet health, immunity and protein digestibility; reduce diarrhea rate and mortality; and positively regulate gut microbiota for nutrient metabolism and to promote healthy conditions.

## Figures and Tables

**Table 1:** Compositions and nutrient levels of basal diet (%).

Items	Content	Items	Content
Diet compositions			
Soybean oil	2.0	Acidulant	0.1
Corn meal	50.5	Lysine	0.5
Wheat flour	13.0	Methionine	0.25
Riedel wheat	0.5	Threonine	0.15
Puffed soybean	6.0	Tryptophan	0.03
Soybean meal	18.0	Stone powder	0.47
Sugar	1.0	Calcium hydrophosphate	1.0
Glucose	0.5	Montmorillonite	0.4
Salt	0.3	Premix	5.0
Citric acid	0.3	Total	100
Nutrient levels			
CP	19.79	Met + Cys	0.86
DE (MJ/Kg)	12.86	Ca	0.61
Lys	1.38	TP	0.59
Met	0.57	AP (Available P)	0.36

Note: Crude protein (CP), calcium (Ca) and total phosphorus (TP) contents were measured, while the other nutrient contents were calculated. Premix (per Kg of diet) provides: Cu 2597 mg; Fe 2945 mg; Zn 2665 mg; Mn 1190 mg; I 197 mg; Se 197 mg; VA 29,400 IU; VD_3_ 2200 IU; VE 1650 IU; VK 1.03 mg; VB1 0515 mg; VB2 14.7 mg; VB12 61.8 μg; pantothenic acid 32.96 mg; nicotinic acid 61.8 mg; choline 125 mg; folic acid 1.03 mg; biotin 0.21 mg.

**Table 2:** Effect of CPB on piglet growth performance.

Items	A	B	C	D	E
Initial weight (kg)	9.63 ± 0.86	9.60 ± 0.83	9.56 ± 0.81	9.55 ± 0.90	9.58 ± 0.57
Final weight (kg)	25.17 ± 1.67	25.12 ± 1.51	23.77 ± 3.10	23.95 ± 1.22	26.61 ± 1.40
ADG (g)	370.04 ± 29.78	369.48 ± 26.85	359.82 ± 95.60	336.90 ± 21.91	405.51 ± 44.76
ADFI (g)	608.26 ± 0.03	625.32 ± 0.08	590.61 ± 0.05	574.27 ± 0.07	602.06 ± 0.05
FCR	1.64 ± 0.21	1.69 ± 0.11	1.64 ± 0.22	1.70 ± 0.06	1.48 ± 0.13
Diarrhea rate (%)	2.19	1.11	1.69	1.81	1.66
Mortality (%)	5.00	3.75	1.88	2.50	3.75
Rejection rate (%)	7.50	0.00	0.00	0.00	0.00

Note: The data are shown as means ± SEM (n = 4). The different lowercase letters in the same rows indicate significant difference (*p* < 0.05), while the same or without lowercase letters in the same rows indicate insignificant difference (*p* > 0.05). A: basal diet; B: basal diet supplemented with antibiotics and zinc oxide; C, D and E: basal diets supplemented with 0.06%, 0.12% and 0.18% compound probiotics and berberine (CPB), respectively. The rejected piglets for “Rejection rate” calculation indicate that the serious weak and sick piglets are removed from the experiment for special care in order to reduce mortality according the pig farm rule.

**Table 3:** Effect of CPB on piglet nutrient digestibility (%).

Group	CP	EE	P	Ca
A	69.63 ± 0.15 ^b^	66.49 ± 2.95	78.19 ± 0.44	68.83 ± 0.22
B	71.87 ± 0.84 ^b^	70.87 ± 4.59	77.57 ± 0.48	67.51 ± 0.73
C	79.43 ± 1.57 ^a^	72.66 ± 4.29	76.38 ± 1.51	67.44 ± 0.08
D	79.25 ± 1.01 ^a^	68.41 ± 3.19	77.21 ± 0.29	66.48 ± 1.18
E	80.66 ± 1.95 ^a^	65.32 ± 1.29	78.28 ± 0.08	67.21 ± 0.88

Note: The data are shown as means SEM (n = 5). The different lowercase letters in the same columns indicate significant difference (*p* < 0.05), while the same or without lowercase letters in the same columns indicate insignificant difference (*p* > 0.05). A: basal diet; B: basal diet supplemented with antibiotics and zinc oxide; C, D and E: basal diets supplemented with 0.06%, 0.12% and 0.18% CPB, respectively. CP: crude protein; EE: ether extract; P: phosphorus; Ca: calcium.

**Table 4:** Effect of CPB on serum indexes of piglets.

Items	A	B	C	D	E
UN (mmol/L)	3.79 ± 0.79 ^ab^	5.31 ± 1.00 ^a^	3.38 ± 0.38 ^b^	3.16 ± 0.42 ^b^	4.57 ± 1.80 ^ab^
GLU (mmol/L)	5.40 ± 0.41	6.27 ± 0.86	4.94 ± 0.74	5.71 ± 1.27	5.13 ± 0.71
TP (g/L)	59.78 ± 3.54	58.75 ± 3.34	59.15 ± 3.97	64.40 ± 4.76	62.48 ± 3.75
IgM (g/L)	0.35 ± 0.18	0.20 ± 0.04	0.26 ± 0.12	0.22 ± 0.03	0.31 ± 0.07
IgG (g/L)	3.27 ± 0.54 ^b^	4.23 ± 0.74 ^ab^	4.74 ± 0.56 ^ab^	5.10 ± 1.72 ^a^	4.78 ± 1.32 ^ab^
ALT (U/L)	113.25 ± 43.41 ^a^	47.50 ± 8.35 ^b^	75.50 ± 14.84 ^ab^	72.67 ± 12.10 ^ab^	78.40 ± 31.73 ^ab^
AST (U/L)	74.75 ± 17.58 ^ab^	51.75 ± 8.26 ^b^	80.25 ± 22.20 ^a^	71.33 ± 11.02 ^ab^	62.80 ± 17.56 ^ab^
ALP (U/L)	382.50 ± 57.91 ^a^	187.00 ± 51.62 ^b^	266.75 ± 94.95 ^ab^	318.00 ± 130.23 ^ab^	202.00 ± 72.36 ^b^

Note: The data were shown as means ± SEM (n = 3). The different lowercase letters in the same rows indicate significant difference (*p* < 0.05), while the same or without lowercase letters in the same rows indicate insignificant difference (*p* > 0.05). UN: urea nitrogen; GLU: glucose; TP: total protein; IgM: immunoglobin M; IgG: immunoglobin G; ALT: alanine aminotransferase; AST: aspartate aminotransferase; ALP: alkaline phosphatase; A: basal diet; B: basal diet supplemented with antibiotics and zinc oxide; C, D and E: basal diet supplemented with 0.06%, 0.12% and 0.18% CPB, respectively.

**Table 5:** Prediction for KEGG pathways of bacterial communities

Items	A	B	C
Cellular processing	347977	462088	254405
Environmental information processing	1277640	1502226	1031051
Genetic information processing	2262700	2662893	2076586
Human diseases	78762	93508	74579
Metabolism	5018673	6010831	4714755
None	20708	26475	19759
Organismal systems	84390	102617	77360
Unclassified	1439542	1725852	1354165

Note: group A: basal diet; group B: basal diet supplemented with antibiotics and zinc oxide; group C: basal diet supplemented with 0.06% CPB.

## Data Availability

The raw reads of sequencing data in this study have been uploaded to SRA (Sequence Read Archive) of NCBI under the accession number of SRP196521.
